# Is there a link between non-alcoholic fatty liver disease aspects and pancreatic cancer? Results of a case-matched study

**DOI:** 10.1590/0100-6991e-20202913

**Published:** 2021-06-28

**Authors:** ACHILES QUEIROZ MONTEIRO DE REZENDE, MARTINHO ANTÔNIO GESTIC, MURILLO PIMENTEL UTRINI, FELIPE DAVID MENDONÇA CHAIM, HELENA PAES DE ALMEIDA DE SAITO, ELINTON ADAMI CHAIM, FRANCISCO CALLEJAS-NETO, EVERTON CAZZO

**Affiliations:** 1 - Universidade Estadual de Campinas, Departamento de Cirurgia - Campinas - SP - Brasil

**Keywords:** Non-alcoholic fatty liver disease, Fatty liver, Pancreatic neoplasms, Carcinogenesis, Carcinoma, Pancreatic Ductal, Hepatopatia Gordurosa não Alcoólica, Fígado Gorduroso, Neoplasias Pancreáticas, Carcinogênese, Carcinoma Pancreático Ductal

## Abstract

**Background and Aims:**

An association between non-alcoholic fatty liver disease (NAFLD) and pancreatic ductal adenocarcinoma (PDAC) has been previously suggested. This study aims at investigating this association and at identifying potential links between variables of the NAFLD spectrum and PDAC.

**Methods:**

A cross-sectional case-matched analytical and comparative study was carried out to analyze patients undergoing surgical resection of PDAC and compare them to a control group of individuals undergoing cholecystectomy at a public tertiary teaching hospital, matched by sex, age and BMI. Hepatic histopathological examinations were compared between cases and controls*.*

**Results:**

Of 56 individuals, 36 were male (64.3%) and the median age was 61.5 years old (interquartile range: 57.5 - 70). The participants’ median BMI was 24.3 kg/m^2^ (interquartile range: 22.1-26.2 kg/m^2^). Microvesicular steatosis (p=0.04), hepatocellular ballooning (p=0.02), fibrosis (p=0.0003) and steatohepatitis (p=0.03) were significantly more frequent in the group of cases. Odds ratios for hepatocellular ballooning (6.2; 95%CI: 1.2-31.8; p=0.03), fibrosis (9.3; 95%CI: 2.5-34.1; p=0.0008) and steatohepatitis (3.9; 95%CI: 1.1-14.3; p=0.04) were statistically significant in relation to the PDAC prevalence.

**Conclusions:**

Significant associations were identified between histopathological aspects of NAFLD (microvesicular steatosis, hepatocellular ballooning, fibrosis, and steatohepatitis) and PDAC.

## RESUMO

**Histórico e objetivos:**

a associação entre a doença hepática gordurosa não alcoólica (DHGNA) e o adenocarcinoma ductal pancreático (ACDP) foi sugerida anteriormente. Este estudo visa investigar esta associação e identificar possíveis ligações entre as variáveis do espectro da DHGNA e o ACDP.

**Métodos:**

foi realizado estudo transversal caso-controle analítico e comparativo para analisar pacientes submetidos a ressecção cirúrgica de ACDP e compará-los a grupo controle de indivíduos submetidos a colecistectomia em hospital público terciário de ensino, pareados por sexo, idade e IMC. Os exames histopatológicos hepáticos foram comparados entre casos e controles.

**Resultados:**

dos 56 indivíduos, 36 eram do sexo masculino (64,3%) e a idade mediana era de 61,5 anos de idade (intervalo interquartil 57,5-70). A mediana do IMC dos participantes foi de 24,3 kg/m^2^ (intervalo interquartil 22,1 26,2). Esteatose microvesicular (p = 0,04), balonização hepatocelular (p = 0,02), fibrose (p = 0,0003) e esteato-hepatite (p = 0,03) foram significativamente mais frequentes no grupo de casos. As razões de chances para balonização hepatocelular (6,2; IC 95%: 1,2 - 31,8; p = 0,03), fibrose (9,3; IC 95%: 2,5 - 34,1; p = 0,0008) e esteato-hepatite (3,9; IC 95%: 1,1 - 14,3; p = 0,04) foram estatisticamente significativas em relação à prevalência de ACDP.

**Conclusões:**

houve associações significativas entre aspectos histopatológicos de DHGNA (esteatose microvesicular, balonização hepatocelular, fibrose e esteato-hepatite) e a ocorrência de ACDP.

## INTRODUCTION

Non-alcoholic fatty liver disease (NAFLD) is currently the most common liver disease worldwide, affecting about 25% of the population. It ranges from simple hepatic steatosis - of which at least 5% of hepatocytes present with fat deposition - to more severe forms, such as non-alcoholic steatohepatitis (NASH), defined as steatosis associated with lobular inflammation and hepatocellular ballooning, and fibrosis, characterized by regenerative abnormalities that can lead to the disruption of the liver cyto-architecture (cirrhosis). NAFLD-associated cirrhosis is associated with a high risk of developing hepatocellular carcinoma^1^ ^,^ ^2^.

Studies on the association between NAFLD and extrahepatic neoplasms were conducted with a focus on several neoplasms of the digestive tract. The most representative data are focused on colorectal cancer, perhaps due to its higher incidence, but studies investigating an association with gastric, esophageal and pancreatic neoplasms have also been performed^2^. Wong et al., in a study that correlated findings of colorectal neoplasms found at colonoscopy and NAFLD diagnosis through nuclear magnetic resonance by spectroscopy and liver biopsy, observed a higher incidence of polyps and colorectal neoplasm, especially in the group of patients with NASH compared to the control group of healthy individuals^3^. 

NAFLD and visceral adipose tissue are the main components of the central obesity axis with importance in carcinogenesis. In this scenario, chronic low-grade inflammation and insulin resistance create a suitable microenvironment for the development of cancer by stimulating the insulin growth factor-1 (IGF-1) axis through hyperinsulinemia. Through its proliferative and anti-apoptotic effects, this pathway can drive mutations that favor carcinogenesis^2^. With the increase in the accumulation of fatty acids and triglycerides, hepatic gluconeogenesis is activated, leading to the release of a series of inflammatory cytokines, which under abnormal levels can induce anti-apoptotic effects, cell proliferation, angiogenesis and invasiveness of cancer cells. Adiponectin has anticarcinogenic effects by directly inhibiting tumor necrosis factor-α (TNF-α) and stopping the activation of cancer cell caspase. However, in obese patients with NAFLD, there is a decrease in the levels of adipocytokine and an increase in leptin, another mediator that has an antagonistic effect, being associated with the maintenance of a pro-inflammatory environment, favorable to tumor development, and being also related to effects that promote cancer progression, including angiogenesis, cell migration and mitogenesis^4^. 

Another pathway of critical relevance related to NAFLD and carcinogenesis, including pancreatic cancer, is the hedgehog pathway (“hedgehog signaling pathway”), which is present in the tissue repair process and is activated in chronic hepatic inflammatory processes. It has also been evidenced as active in pancreatic precursor lesions such as pancreatic intraepithelial neoplasm (PanIN) and already degenerated ductal adenocarcinomas. Once active, it provides cellular activity, and it has been reported that hedgehog pathway activation and KRAS mutation together can perform cell initiation in pancreatic cancer^5^. 

The determination of a potential association between NAFLD and the components of its histopathological spectrum with pancreatic ductal adenocarcinoma (PDAC) would be of great use in clinical practice, since NAFLD would constitute a potential modifiable and highly prevalent risk factor, which would open possibilities for screening and primary/secondary prevention strategies aimed at its occurrence.

The current study aims at analyzing the existence of a potential association between NAFLD assessed through histopathological examinations and PDAC in a controlled study, and at identifying associations between the variables of the NAFLD histopathological spectrum and PDAC.

## METHODS

### Study Design

A cross-sectional case-matched analytical and comparative study was carried out to analyze patients undergoing surgical resection of PDAC and compare them to a control group of individuals undergoing cholecystectomy in a public tertiary teaching hospital, matched by sex, age and BMI, between January 2018 and December 2019. 

### Sampling Calculation

The sample size of the current study was calculated based on the hypothesis of a two-sided equity for cross-sectional studies with paired groups as proposed by Dupont^6^. The following parameters were considered: probability of type I error (α) of 5%, probability of error type II (β) of 20% and odds ratio for the risk factor (NAFLD) of 5. The minimum sample calculated was 50 individuals (25 in each group).

### Study Population

Study participants were identified using the electronic medical record system. Individuals undergoing surgical PDAC resection were selected for the case group during the considered period. The individuals that compose the control group were selected from a database of patients who underwent cholecystectomy at the same facility in the same period, matched according to sex, age and body mass index (BMI).

Inclusion criteria were: patients of any sex; age older than 18 years; patients with PDAC undergoing surgical resection with a diagnosis confirmed by histopathological examination (cases); or undergoing cholecystectomy (controls). The exclusion criteria were: patients with incomplete medical records; cases with a histopathological diagnosis other than PDAC; control group patients with active malignancy from any primary site; active cholestasis according to laboratory tests at the time of the procedure; clinical jaundice reported at the time of the procedure; significant active or prior alcoholism; previous chronic liver diseases other than NAFLD; liver biopsy not performed; vulnerable groups (younger than 18 years old, mentally handicapped, institutionalized).

**Figure 1:**
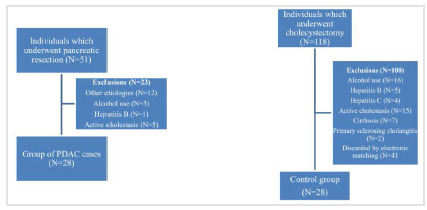
Flowchart of the study population. PDAC: pancreatic ductal adenocarcinoma.

There were 39 individuals who underwent pancreatic resection due to neoplasms and 118 cholecystectomies in the considered period. After applying the exclusion criteria and matching by sex, age, and BMI, 56 individuals were selected for the study (28 in each group). Figure 1 shows a flowchart of the study population.

### Liver Biopsy

A wedge liver biopsy was performed on all individuals included in the study, following a standardized method. A 2-cm fragment was extracted from segment III of the left hepatic lobe with Metzebaum scissors at the end of the surgical procedures. After extraction, hemostasis was performed with electrocautery.

### Variables

The analyzed variables were age, sex, BMI, presence of comorbidities, and liver biopsy findings, which were classified according to the current protocol of the Brazilian Society of Hepatology that analyzes various parameters from a semi-quantitative and topological point of view. Histological parameters were categorically assessed as follows: macrovesicular steatosis, microvesicular steatosis; hepatocellular ballooning; acinar and/or portal inflammation; and perisinusoidal and/or periportal fibrosis. These features were dichotomously classified as present or absent^7^ ^,^ ^8^. The findings of the hepatic histopathological examination were compared between the groups of cases and controls.

### Statistical analysis

Descriptive analysis is presented with frequency tables for categorical variables and measures of position and dispersion for numerical variables. Normality was assessed using the Shapiro-Wilk test. The pairing was performed by electronic selection in a database of individuals undergoing cholecystectomy, with three variables being selected for this purpose (age, sex, and BMI), and it was adopted the 1:1 ratio between cases and controls. To compare proportions, the Chi-square test or Fisher’s exact test was applied when necessary. To compare continuous measures, the Mann-Whitney test was used. To express the ratio between the prevalence of PDAC in both groups, odds ratios and 95% confidence intervals (CI) were calculated. The level of significance adopted for the statistical tests was 5% (p <0.05). The SAS System for Windows (Statistic Analysis System), version 9.2, was used to perform the analysis and pairing; SAS Institute Inc., 2002-2008, Cary, NC, USA.

## RESULTS

Of the 56 selected individuals, 36 were male (64.3%) and the median age was 61.5 years (interquartile range: 57.5-70). The participants’ median BMI was 24.3 kg/m2 (interquartile range: 22.1-26.2). There were 25 individuals with diabetes (44.6%), 21 with hypertension (37.5%) and 8 with dyslipidemia (14.3%).

When comparing the groups of cases and controls, there were no statistically significant differences regarding the distribution by sex (p=1.0), age (p=0.9), BMI (p=0.8), and frequencies of diabetes (p=0.4), hypertension (p=0.3) and dyslipidemia (p=0.3). The complete comparison is shown in Table 1.

**Table 1:** Clinical, anthropometric and demographic characteristics of the study population.

	PDAC Cases	Control group	Value of P
N	28	28	NA
Gender	F: 10 (35.7%) M: 18 (64.3%)	F: 10 (35.7%) M: 18 (64.3%)	1.0
Age (years)	63 (IQ: 58.5-68.5)	60 (IQ: 57-71)	0.9
Body mass index (kg/m^2^)	24.2 (IQ: 22.1-25.6)	24.5 (IQ: 22.1-26.8)	0.8
Type 2 Diabetes N (%)	14 (50%)	11 (39.3%)	0.4
Dyslipidemia N (%)	3 (10.7%)	5 (17.9%)	0.3
Hypertension N (%)	9 (32.1%)	12 (42.9%)	0.2

PDAC: pancreatic ductal adenocarcinoma; N: number of individuals; F: female; M: male; IQ: interquartile range.

In the group of cases, it was observed that most tumors were located in the pancreatic head (93%), while the remaining comprised lesions of the body and/or pancreatic tail. Thus, the surgical procedures performed for the resection of these lesions were predominantly pancreato-duodenectomy (93%), while 7% underwent distal pancreatectomy with splenectomy.

Regarding the comparison of the NAFLD-related findings of hepatic histology between the groups of cases and controls, there were statistically significant differences for microvesicular steatosis (p=0.04), hepatocellular ballooning (p=0.02), fibrosis (p=0.0003) and steatohepatitis (p=0.03); all were significantly more frequent in the group of cases. The frequency of macrovesicular steatosis did not differ significantly between the two groups (p=0.4). The frequency of biliary disease related to bile duct obstruction was also significantly higher in the group of cases (p=0.03). Table 2 shows the complete comparison between the hepatic histopathological variables between the groups.

**Table 2:** Comparison of NAFLD-related histopathological aspects between individuals with PDAC and controls.

	PDAC Cases	Control group	Value of P
Macrovesicular steatosis N (%)	13 (46.4%)	10 (35.7%)	0.4
Microvesicular steatosis N (%)	8 (28.6%)	2 (7.1%)	0.04
Hepatocellular ballooning N (%)	9 (32.1%)	2 (7.1%)	0.02
Fibrosis N (%)	17 (60.8%)	4 (14.3%)	0.0003
Steatohepatitis N (%)	11 (39.3%)	4 (14.3%)	0.03

PDAC: pancreatic ductal adenocarcinoma; NAFLD: non-alcoholic fatty liver disease; N: number of individuals.

**Figure 2:**
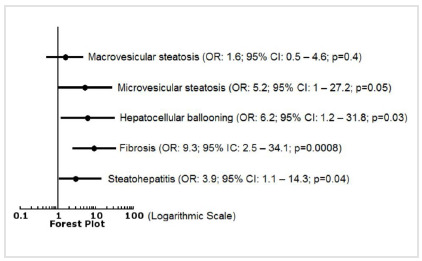
Forest plot describing odds ratios for each histopathological non-alcoholic fatty liver disease-related aspect in relation to pancreatic ductal adenocarcinoma prevalence. OR: odds ratio; CI: confidence interval.

Odds ratios calculated for hepatocellular ballooning (6.2; 95% CI: 1.2-31.8; p=0.03), fibrosis (9.3; 95% CI: 2.5-34.1; p=0.0008) and steatohepatitis (3.9; 95% CI: 1.1-14.3; p=0.04) were statistically significant in relation to the PDAC prevalence. The complete odds ratio analyses are presented in a Forest plot (Figure 2).

## DISCUSSION

In the current study, significant associations were observed between the presence of certain hepatic histopathological aspects (microvesicular steatosis, steatohepatitis, hepatocellular ballooning, fibrosis and biliary hepatopathy) with PDAC, by comparing a group of patients with this neoplasm undergoing surgical resection with a control group of patients undergoing cholecystectomy. Both groups were matched by sex, age, and BMI to minimize potential bias typical of such comparison. It is important to highlight the high frequency of NAFLD in both groups, considering that both had a median BMI below the cutoff value for obesity. There is robust evidence of the association between obesity and NAFLD, but the presence of NAFLD in individuals without obesity cannot be overlooked^4^ ^,^ ^9^. 

Among the histopathological aspects associated with PDAC in the present study, microvesicular steatosis, hepatocellular ballooning, fibrosis, and steatohepatitis represent not only histopathological findings commonly seen in NAFLD, but also disorders considered moderate to severe within the pathological spectrum of this liver disease. Microvesicular steatosis is less common in NAFLD than in alcoholic liver disease and is usually associated with a higher risk of fibrosis and greater difficulty in reversing than macrovesicular steatosis, similarly to hepatocellular ballooning. NASH, on the other hand, represents an important marker of inflammatory activity and worsening insulin resistance involved in the pathogenesis of NAFLD, being one of the factors responsible for the future progression to fibrosis and cirrhosis, and an early indicator of risk of liver carcinogenesis^8^. 

In 2007, the International World Cancer Research Fund determined the association between pancreatic cancer and obesity to be well established. In 2012, a meta-analysis showed an increase in the incidence of this neoplasm according to the growth in the abdominal waist, with an increase in risk of 0.1 for every 10cm^2,^ ^10^. Allen et al., in a longitudinal study, demonstrated a significantly higher incidence of extrahepatic neoplasms in patients with NAFLD regardless of obesity, with an almost 2-fold increase in the absolute risk of extra-hepatic neoplasms, including pancreatic neoplasm^11^. 

Because of the cross-sectional design of the current study, it was not possible to directly analyze the weight trajectory of the group of cases prior to the PDAC diagnosis. Most individuals with PDAC are admitted to the referral service with significant weight loss, often without overweight or obesity. Considering that certain aspects of the histopathological spectrum of NAFLD may improve, or even present complete reversal due to weight loss, the observation of significantly higher frequencies of microvesicular steatosis, steatohepatitis, hepatocellular ballooning and fibrosis is even more relevant, since with a likely massive weight loss before histopathological analysis these individuals maintained these NAFLD-related findings, when in comparison with a control group who did not present weight loss and was matched by sex, age, and BMI. It should also be noted that NAFLD is predominantly a manifestation of intra-abdominal visceral fat deposition over a long period of time and potentially does not present an immediate and directly proportional reduction in weight with the cachexia that these patients present at diagnosis^4^. The findings of the present study reinforce the possibility of an association between NAFLD and PDAC, even in individuals with NAFLD without obesity.

Liver fibrosis, on the other hand, may be associated with NAFLD as part of its spectrum. However, in the context of individuals with PDAC, it may also be related to the previous occurrence of cholestasis secondary to biliary obstruction by the tumor, constituting a result that is difficult to interpret and its meaning should be considered more cautiously. Despite the choice of the current study to exclude patients operated in the presence of cholestasis, there is still some interference of this finding, since fibrosis is not always reversible after the resolution of biliary obstruction, considering that in the group of individuals with PDAC without active cholestasis, the presence of biliary hepatopathy was observed in 70% of cases.

Most of the prior evidence on the association between PDAC and NAFLD is based on population studies in whom NAFLD was assessed using imaging methods, especially ultrasound scan, and in some cases, computed tomography or magnetic resonance^12^. Although these methods have significant accuracy, they are less sensitive and specific than liver biopsy, considered the gold standard, especially in situations where there is less significant deposition of fat in the liver. In addition, these methods are not able to provide a nuanced analysis of the histopathological spectrum of NAFLD, with the evaluation of other variables, such as hepatocellular ballooning and steatohepatitis particularly. Among an extensive literature review carried out by the authors of the current study, there was only a research by Wong et al. who used magnetic resonance imaging and liver biopsy to analyze the association between NAFLD and extrahepatic neoplasm (colorectal neoplasm) in patients undergoing colonoscopy in both groups^3^. The latter authors showed the association of NAFLD and the presence of colorectal disease (adenomatous polyps/advanced neoplasia/dysplasia), which we also observed in our study with a higher incidence of lesions in patients with steatohepatitis^3^. 

Magnetic resonance imaging, with or without spectroscopy, presents adequate sensitivity and specificity for the detection of NAFLD, but it is not used outside the context of clinical studies, due to its cost and availability, in addition to the need for a qualified team to interpret it^13^. Even so, as noted earlier, only biopsy can fully analyze and stratify NAFLD. Thus, the relevance of the findings of the current study cannot be underestimated in regards of previous evidence.

There are studies that have analyzed the association between carcinogenesis and NAFLD assessed by means of non-invasive scores. These scores, calculated from clinical and laboratory data, were developed to assess the degree of liver disease and to detect individuals who would have more precise indications for investigation by complementary exams or even liver biopsy. They present adequate accuracy after population validation, but they must be analyzed with caution in the individual context^14^ ^,^ ^15^. The indices or scores recommended by the American Association for the Study of Liver Diseases (AASLD) for the detection of advanced fibrosis are APRI, FIB-4 and the NAFLD fibrosis score (NFS). It is important to note that these scores were not developed for the screening of incipient fibrosis, detecting only situations where there is fibrosis with the formation of septa (“bridging” fibrosis)^15^. Liu et al., in a meta-analysis that included 26 studies of patient with NAFLD assessed by indirect scores, demonstrated a clear association between NAFLD and extrahepatic neoplasms, including pancreatic cancer, with an odds ratio of 2.12^12^. Kim et al., in a prospective longitudinal study, with a mean follow-up of 7.5 years, also found a significant association between NAFLD, assessed by ultrasound scan and clinical scores for fibrosis and hepatocellular carcinoma, colorectal cancer in men and breast cancer in women. However, they did not show a statistically significant increase in other neoplasms. In that study, a significant association was observed between the presence of FIB-4 and NFS scores greater than 1.45 with the occurrence of liver and extrahepatic neoplasms^16^. Peleg et al., in a retrospective study with an average follow-up of 100 months, also demonstrated that the presence of liver fibrosis detected by biopsy and/or by FIB-4 and NFS were factors independently associated with higher mortality from malignant neoplasms^17^. Insulin resistance, stimulation of insulin-like growth factor (IGF), changes in the microbiome and intestinal permeability, hormonal and inflammatory changes produced by abdominal fat tissue, mediated by the secretion of adipokines and pro-inflammatory mediators, and stimulation of the hedgehog pathways are all potentially interconnected mechanisms in pancreatic carcinogenesis, and are associated with obesity, metabolic syndrome, and NAFLD^18^. 

Thus, it is possible to infer that pancreatic carcinogenesis is complex and multifactorial, with the hepatic manifestation of the metabolic syndrome (NAFLD) being an important component involved in several stages of this process in this group of individuals. The clinical importance of such interrelation between NAFLD and PDAC is of great importance from the moment when a definition of the patients who are part of a group at risk for the neoplasm, as well as in the predisposing genetic syndromes. From the determination of NAFLD and specific components of its histopathological spectrum as risk factors for PDAC, it is possible to define which populations deserve a more rigorous screening for this type of cancer. In addition, it is possible to intensify the introduction of therapeutic measures aiming at the control and resolution of this risk factor, which is modifiable. Considering that, especially in its initial stages, NAFLD is adequately treated by weight loss, which can be achieved with diet, physical activity, psychological treatment, or even with invasive approaches such as bariatric surgery, the adoption of these measures could bring the added benefit of primary prevention of the occurrence of PDAC.

Bariatric surgery has achieved prominence in the last decades, due to its effectiveness in the treatment of obesity and metabolic syndrome as well as its hepatic phenotype, changing the natural evolution of the disease and leading to several secondary benefits, such as improvement of cardiovascular diseases and potentially preventing liver and extrahepatic neoplasms^19^ ^,^ ^20^. Adams et al., in a classic study compared 9,949 obese patients undergoing operation with 9,628 non-operated ones, and they observed a 60% reduction in cancer deaths in the group of operated individuals^21^. Doumouras et al., in a methodologically similar study analyzed two groups of 13,679 individuals with obesity (operated and non-operated). These authors also observed a significantly lower risk of approximately 50% of cancer and cardiovascular mortality in the group of patients undergoing bariatric surgery, after an average follow up of approximately five years^22^. Wiggins et al., in a meta-analysis of population studies that included 635,642 individuals, observed a significant reduction in mortality from neoplasms associated with obesity, among which the authors included the PDAC after bariatric surgery^23^. 

The low overall incidence of PDAC makes it difficult to carry out specific studies, due to the need for exceptionally large population samples to reach statistical significance. Syed et al., analyzing a database of 1,435,350 patients followed for 20 years, demonstrated that obese and diabetic individuals undergoing surgery had a significant reduction in the risk of developing pancreatic cancer in relation to the population who did not undergo bariatric surgery, in the first specific and conclusive evidence on bariatric surgery as a protective factor for PDAC^24^. 

The current study has some limitations that must be taken into consideration. Its small sample size, especially because of various exclusions caused by the presence of active cholestasis, history of alcohol consumption, neoplasms other than PDAC and other chronic liver diseases that could cause bias, is a relevant limiting factor; although appropriate for the scope of the study, this is based on strict population settings, and therefore does not allow further extrapolation of the findings. On the other hand, when choosing a homogeneous sample, the results are relevant within similar populations and are less subject to the effects of confounding variables. Another limitation of this study is its cross-sectional design, which allows associations to be observed, but it’s not ideal to establish causal links. Considering that the previous evidence in the literature points towards the same direction and that the use of histopathological analysis (the gold standard for assessing NAFLD) was unavailable in most other studies, our findings cannot be underestimated. The current study collaborates to increase the understanding of the potential association between NAFLD and pancreatic carcinogenesis, constituting another element in this complex interconnected hypothesis. Further research, with prospective design and larger and more heterogeneous population samples, is necessary to confirm the present findings.

## CONCLUSION

Significant associations were identified between histopathological aspects of NAFLD and the occurrence of PDAC. Significantly higher frequencies of microvesicular steatosis, hepatocellular ballooning, fibrosis, and steatohepatitis were found in the group of individuals with PDAC compared to the control group.
